# 胸腺肿瘤的诊疗：基于中国胸腺肿瘤协作组多中心回顾性研究的共识

**DOI:** 10.3779/j.issn.1009-3419.2016.07.02

**Published:** 2016-07-20

**Authors:** 文涛 方, 剑华 傅, 毅 沈, 煜程 魏, 黎杰 谭, 鹏 张, 泳涛 韩, 椿 陈, 仁泉 张, 印 李, 克能 陈, 和忠 陈, 永煜 刘, 有斌 崔, 允 王, 烈文 庞, 振涛 于, 鑫明 周, 阳春 柳, 刚 陈, 中国胸腺肿瘤协作组成员

**Affiliations:** 1 200030 上海，上海交通大学附属上海胸科医院 Department of Thoracic Surgery, Shanghai Chest Hospital, Shanghai Jiao Tong University, Shanghai 200030, China; 2 510060 广州，中山大学附属肿瘤医院胸外科 Department of Thoracic Surgery, Guangdong Esophageal Cancer Institute, Sun Yat-sen University Cancer Center, State Key Laboratory of Oncology in South China, Collaborative Innovation Center of Cancer Medicine, Guangzhou 510060, China; 3 266001 青岛大学医学院附属医院胸外科 Department of Thoracic Surgery, Afliated Hospital of Qingdao University, Qingdao 266001, China; 4 200032 上海，复旦大学附属中山医院胸外科 Department of Thoracic Surgery, Zhongshan Hospital, Fudan University, Shanghai 200032, China; 5 300052 天津，天津医科大学附属总医院胸外科 Department of Endocrinology, Tianjin Medical University General Hospital, Tianjin 300052, China; 6 610041 成都，四川省肿瘤医院胸外科 Department of Thoracic Surgery, Sichuan Cancer Hospital, Chengdu 610041, China; 7 350001 福州，福建医科大学附属协和 医院胸外科 Department of Thoracic Surgery, Fujian Medical University Union Hospital, Fuzhou 350001, China; 8 230022 合肥，安徽医科大学附属第一医院胸外科 Department of Thoracic Surgery, First Afliated Hospital of Anhui Medical University, Hefei 230022, China; 9 450008 郑州，郑州大学附属肿瘤医院胸外科 Department of Thoracic Surgery, Affiliated Cancer Hospital of Zhengzhou University, Zhengzhou 450008, China; 10 100142 北京，北京大学附肿瘤医院胸外科 Department of Thoracic Surgery, Beijing Cancer Hospital, Beijing 100142, China; 11 200433 上海，长海医院胸心外科 Department of Cardiothoracic Surgery, Changhai Hospital, Shanghai 200433, China; 12 110042 沈阳，辽宁肿瘤医院胸外科 Department of Thoracic Surgery, Liaoning Cancer Hospital, Shenyang 110042, China; 13 130021 长春，吉林大学附属第一医院胸外科 Department of Thoracic Surgery, First Afliated Hospital of Jilin University, Changchun 130021, China; 14 610041 成都，四川大学华西医院胸外科 Department of Thoracic Surgery, West China Hospital, Sichuan University, Chengdu 610041, China; 15 200032 上海，复旦大学附 属华山医院胸外科 Department of General Surgery, Huashan Hospital, Fudan University, Shanghai 200032, China; 16 300060 天津，天津医科大学附属肿瘤医院食管癌中心 Department of Esophageal Cancer, Tianjin Cancer Hospital, Tianjin 300060, China; 17 310022 杭州，浙江省肿瘤医院胸外科 Department of Thoracic Surgery, Zhejiang Cancer Hospital, Hangzhou 310022, China; 18 330006 南昌，江西省人民医院胸外科 Department of Thoracic Surgery, Jiangxi People's Hospital, Nanchang 330006, China

胸腺肿瘤是胸部实体肿瘤中相对罕见的一个类型^[1]^。在中国，胸腺肿瘤的发病率约为3.93/100万，这大致为肺癌发病率的1/100、食管癌发病率的1/25。此发病率高于北美报告的胸腺肿瘤发病率[2.14/100万；基于美国医疗保险监督、流行病学和最终结果（Surveillance, Epidemiology, and End Results, SEER）数据库]。不过，SEER数据库也显示，胸腺肿瘤在亚裔中的发病率（3.74/100万）要远高于白人（1.89/100万)，且与中国的数据接近。这意味着，胸腺肿瘤在不同的种族和种属之间可能存在差异。与此同时，这两个登记系统均只记录了临床上已处于进展期的“恶性肿瘤”；许多尚处于早期的低级别病变被视作“良性肿瘤”，故未予登记。因此，胸腺肿瘤的确切发病率被大大低估了。随着其他恶性肿瘤（如肺癌）的筛查日益推广，更早期的胸腺肿瘤也有望被及早发现。

事实上，目前所有胸腺肿瘤均已被视作恶性肿瘤^[2]^。现已证实，即使A型胸腺瘤也可存在远处转移，并且A型胸腺瘤完全切除后出现复发也时有报道。因此，“恶性”或“良性”胸腺瘤的区分不再适用，相关的术语也已不再适宜。建议采纳国际胸腺肿瘤协作组织（International Thymic Malignancy Interest Group, ITMIG）提出的“胸腺恶性肿瘤”（Thymic Malignancies）这一术语。同时，该病属于一种惰性肿瘤；在许多胸腺瘤患者中，即便疾病进展后，患者的生存期仍较长。因此，建议针对胸腺肿瘤开展较长时间的随访（10年），着重了解患者总生存率（overall survival, OS）和复发状况^[3]^。

鉴于该肿瘤较罕见且相对惰性，故极难开展大规模的前瞻性随机研究，也很难获得高质量证据来指导临床实践。因此，目前针对胸腺肿瘤的诊治尚存在诸多争议。目前广为采用的胸腺瘤Masaoka分期系统是30多年前根据单个机构不足100个病例的数据而提出的^[4]^；世界卫生组织（World Health Organization, WHO）提出的组织学分类一直饱受争议，即使目前它已获得了越来越广泛的认可^[5]^；目前可用的临床指南一般是根据专家意见或单中心回顾性研究的结果而制订的。因此，当前亟需推动和加强全球性或区域性的协作，以改变当前的局面。ITMIG成立于2010年，致力于胸腺恶性肿瘤的研究、教育和对患者提供支持。全球已有数以百计的成员加入了该组织。为积极响应ITMIG的号召，中国胸腺肿瘤协作组（Chinese Alliance of Research for Thymomas, ChART）于2012年成立。在来自14个省、直辖市的18所三级诊疗中心的大力支持下，ChART已成功建立了首个全国性的胸腺恶性肿瘤数据库。该数据库迄今已收录了1994年-2012年间诊治的2, 500例胸腺肿瘤，并且，已经对胸腺肿瘤的临床病理特征、诊疗模式和患者转归展开回顾性分析，并通过对过去两个10年间（1994年-2003年和2004年-2012年）相关诊疗情况的比较，研究人员也对胸腺肿瘤的诊断、治疗、预后的变化进行了分析。这些结果已集中刊登在《*Journal of Thoracic Diseases*》（胸部疾病杂志）。我们有理由相信，关于胸腺肿瘤的ChART共识可以向未来的临床实践和研究工作提供参考。

胸腺肿瘤的一个独特的特点是其常可伴发自身免疫性疾病，尤其是重症肌无力（myasthenia gravis, MG）（可见于22.8%的患者；基于ChART数据库）。超过90%的重症肌无力患者含有B型胸腺瘤成分。同期伴发的MG症状常有助于对肿瘤的早期检测。2/3伴有MG的胸腺肿瘤患者术后病理分期为Ⅰ期和Ⅱ期肿瘤；即便是在进展期（Ⅲ期和Ⅳ期）肿瘤中，伴有MG的患者组织学类型的恶性程度较低（胸腺瘤，而非胸腺癌或胸腺类癌）。这有助于解释为何伴发MG的患者具有显著较佳的10年OS。不过，不伴发MG的Ⅰ期胸腺肿瘤患者具有更好的生存状况，表明MG仍为一种不良预后因素^[6]^。

目前手术仍为胸腺肿瘤最常使用的治疗方式，藉此实现治愈的机会也最大^[7]^。在ChART回顾性数据库中，仅5.5%的患者接受了非手术治疗。单纯手术切除更常用于病变早期阶段（Ⅰ期：69.9%；Ⅱ期：55.3%，Ⅲ期：23.6%；Ⅳ期：21.5%）以及低度恶性肿瘤（胸腺瘤：53.2% *vs*胸腺癌：20.1%；*P* < 0.001）。总体而言，胸腺肿瘤经手术治疗后的转归在过去20年间已有大幅改善。这一点在ChART回顾性数据库中反映得很明显：总体切除率上升（82.1% *vs* 88.1%），尤其是在胸腺癌患者（62% *vs* 83.3%, *P* < 0.05）和Ⅲ期胸腺瘤患者中（73.9% *vs* 89.5%, *P* < 0.05）。其次，胸腺手术中微创手术的使用率也有所上升，尤其是针对早期疾病。在过去的10年间，电视辅助胸腔镜手术（video-assisted thoracoscopic surgery，VATS；含机器人手术）已在Ⅰ期和Ⅱ期胸腺肿瘤的手术治疗中占据1/4的比例，并在2010年后攀升至4 0%以上。对于病理分期为Ⅰ期和Ⅱ期胸腺肿瘤，VATS切除后的5年OS与开胸手术相近，这提示VATS可实现与传统开胸手术相当的远期转归^[8]^。

在行胸腺肿瘤的外科治疗时，何为适宜的切除范围一直存在争议。胸腺切除术和肿瘤切除术（部分胸腺切除术或胸腺瘤切除术）已在中国广泛应用；其中，在对早期病变行微创手术时，胸腺瘤切除术应用得较多。在本组病例中，超过2/3的Ⅰ期和Ⅱ期患者接受了胸腺切除术。虽然接受胸腺切除术或胸腺瘤切除术两组患者的总体上生存差异不大，但是对于Ⅱ期胸腺肿瘤患者，其胸腺切除组的OS要高于胸腺瘤切除术组，且复发率显著较低。另外，对于伴发MG的患者，胸腺切除术组的术后缓解率也要高于胸腺瘤切除术组^[9]^。这些结果表明，在对胸腺恶性肿瘤行手术治疗时，应优先考虑胸腺切除术作为标准术式，即便肿瘤尚处于早期。

需要再次强调的是，完整切除对于胸腺肿瘤的预后至关重要。所以，术前检查时既要关注肿瘤的分期状况，也要判断肿瘤是否能够完整切除。在多变量分析中，虽然计算机断层扫描（computed tomography, CT）所见[如肿瘤形状、轮廓、强化、是否侵及邻近结构（纵隔脂肪、纵隔胸膜、肺、心包、纵隔血管、膈神经）、是否存在胸膜/心包积液或肺内转移]均与Masaoka-Koga分期存在关联，但只有“未侵及动脉系统”提示原发病变可完整切除^[10]^，这一结果与ITMIG提出的新分期系统^[11]^相呼应。在该新分期系统中，肿瘤侵及动脉系统或心包内血管结构应被视作T_4_期，已不适合首先接受手术治疗。

与胸腺瘤相比，胸腺癌更多地采取综合治疗。这些治疗方法包括辅助放疗（58.9% *vs* 38.3%, *P* < 0.001）、化疗（37.2% *vs* 8.6%, *P* < 0.001）、术前诱导治疗（8.7% *vs* 3.5%, *P* < 0.001）；对于非手术患者，则采取针对性放/化疗。为改善进展期胸腺肿瘤的转归，提高肿瘤完整切除率至关重要。虽然本组中仅有不到5%的患者接受了诱导疗法，但在接受新辅助治疗的患者中有高达25%的患者实现了肿瘤降期和完整切除率的提高^[12]^。对于术前判断可能不能获得手术完整切除的肿瘤，经过有效的新辅助疗法后，局部进展期的肿瘤有望获得降期，其手术切除率和生存状况可能并不劣于那些被认定可切除（进而直接接受手术）的肿瘤；此外，其手术完整切除率和生存状况也显著优于经诱导治疗后无效的肿瘤。对于临床判断不能手术完整切除的胸腺肿瘤或临床上不能手术的患者，越来越多的证据表明同步放化疗可能要比序贯放化疗或单纯化疗更有利于患者预后^[1]^。值得一提的是，治疗前行组织学诊断的比例在过去的20年间已显著上升（11.8% *vs* 18.6%, *P*=0.008）。对于Ⅲ期和Ⅳa期胸腺肿瘤而言，根治性切除率在先行诱导疗法再手术的患者中要显著高于直接接受手术的患者。在诱导疗法后实现肿瘤降期的患者中，其OS似乎高于直接接受手术的患者^[14]^。不过，如肿瘤经新辅助疗法治疗后无效，则患者预后仍然较差，甚至可能比接受针对性放化疗的患者更差。显然，未来需要更加关注那些有效的新辅助疗法，以改善进展期胸腺肿瘤的转归。

总体而言，中国治疗胸腺恶性肿瘤的远期预后与世界各地报告的数据是相似的。随访结果表明，本组患者的5年和10年OS分别为85.3%和76.4%。手术切除后仅17%的肿瘤出现复发，且临床分期越靠后，肿瘤的复发率相对越高（Ⅰ期：3.1%；Ⅱ期：7.3%；Ⅲ期：30.7%；Ⅳ期：48.5%）；此外，病理学恶性程度越高，复发率也更高（A/AB型：2.9%；B1-3型：14.9%；C型：39.7%）。多因素分析显示，肿瘤分期、组织学类型和切除状态均为独立预后因素，而是否合并MG或辅助治疗方式则与生存状况的改善无关。这是根据多个大型单中心队列研究最常报告的结果而得出的结论^[15]^。在近20年间，中国的胸腺肿瘤诊疗已有了显著改善。这主要反映在总复发率的下降（25.4% *vs* 14.5%, *P* < 0.05），特别是在B型胸腺瘤和胸腺癌患者中。虽然OS未见显著差异（82.7% *vs* 85%, *P*=0.618），但胸腺癌患者的生存率已呈现上升趋势，尤其是在Ⅲ期患者中（45.8% *vs* 60.7%, *P*=0.077）。随着单纯手术在胸腺恶性肿瘤中的应用日益增加，手术后辅助治疗的应用越来越少，尤其是在早期肿瘤和低度恶性肿瘤患者中。与单纯手术相比，完整切除后行辅助放疗并不能改善Ⅰ期-Ⅲ期胸腺肿瘤患者的生存状况。不过，在未获完整切除的患者，增加术后放疗确可改善远期预后^[16]^。同样，在Ⅲ期-Ⅳa期胸腺瘤或胸腺癌患者中行辅助化疗并不能改善生存^[17]^。在过去的二十年间，胸腺肿瘤的诊疗方式和预后都有了很大的改变；即便不行辅助放疗，Ⅰ期和Ⅱ期肿瘤患者的生存状况仍令人满意，这可能是因为早期病变的完整切除率较高。鉴于辅助疗法并无明显改进，Ⅲ期胸腺癌患者生存率的提高和复发率的下降主要归功于手术切除率的提高。就Ⅲ期胸腺瘤而言，生存率和复发率保持不变，与之相对应的则是切除率的提高和辅助放疗比例的下降。所有这些都表明：术后放疗对于早期肿瘤而言可能是不必要的，因为这些肿瘤可获得完整切除，且在完整切除后很少复发。Ⅲ期胸腺瘤和胸腺癌接受辅助放疗后是否可以获益，尚需进一步评估。

总之，胸腺恶性肿瘤是一组较为罕见的惰性肿瘤，具有独特的临床病理特点。从使用ChART数据库得到的一系列回顾性研究的结果来看，可达成以下共识，藉以指导未来的研究和临床实践：

① 所有胸腺肿瘤均为恶性，尽管从组织学和临床表现而言多数胸腺肿瘤的恶性程度相对较低。诊治胸腺肿瘤时既要避免治疗过度也要避免治疗不足。

② 在制订治疗方案时，应充分考虑肿瘤的分期和组织学类型。在术前、术后决策时必须有多学科的专业人员参与。

③ 应尽力实现根治性切除；如肿瘤可完整切除，则可望实现最佳预后。为此，在通过影像学检查行治疗前评估时，既要注重肿瘤分期，也要充分考虑肿瘤的可完整切除性。

④ 对于早期肿瘤，单纯手术即可；尚无证据支持在肿瘤完整切除后行术后辅助治疗。

⑤ 微创手术是安全的，且在技术上可行；因此，对于早期肿瘤应尝试应用微创手术。虽然微创手术的围术期结果可优于开胸手术，但仍需开展进一步的研究以探讨其远期疗效。

⑥ 虽然目前尚无定论，但仍推荐行全胸腺切除术以确保实现完整切除，并降低术后复发风险。在切除肿瘤时，完整切除前纵隔脂肪组织可在伴发重症肌无力的胸腺瘤患者中实现较佳结果。

⑦ 重症肌无力作为胸腺恶性肿瘤的常见伴发疾病，常表现为低度恶性的组织学类型，并有助于及早发现胸腺肿瘤。在此基础上，可望实现较高的手术切除率和更佳的生存状况；不过，早期肿瘤伴发的重症肌无力本身可导致死亡率升高，由此在一定程度上可抵消上述优势。

⑧ 对于已处于进展期的恶性程度较高的胸腺肿瘤，有可能通过综合治疗实现较好的预后；特别是通过准确的术前分期、组织学诊断和有效的诱导治疗（以期获得降期），来增加肿瘤完整切除的机会。

⑨ 常规应用辅助放疗和传统化疗药物的效果一直不能令人满意。应注意选择那些复发风险高、而更有望从辅助疗法中获益的患者进行术后辅助治疗。应针对胸腺肿瘤进一步探讨更有效的治疗方式并研发新的药物。

⑩ 对于不能手术切除的胸腺肿瘤或临床上无法手术治疗的患者，同步化放疗有望实现更好的疾病控制并延长生存。

目前，胸腺恶性肿瘤的诊疗仍有许多问题有待解决。鉴于胸腺肿瘤较罕见且相对惰性，在临床研究中应加强协作，以更深入地认知这一疾病。与ITMIG全球数据库项目一样，ChART回顾性数据库分析也为罕见肿瘤（如胸腺疾病）的研究树立了一个良好榜样^[3]^。当前亟需加强不同区域间多个中心之间的协作，以组织大规模的临床研究来解决现有的问题，并为进一步改进临床实践铺平道路。
